# Reactogenicity of BNT162b2 mRNA COVID-19 Vaccine in a Young Working Age Population: A Survey among Medical School Residents, within a Mass Vaccination Campaign, in a Regional Reference Teaching Hospital in Italy

**DOI:** 10.3390/vaccines9111269

**Published:** 2021-11-03

**Authors:** Alborz Rahmani, Guglielmo Dini, Andrea Orsi, Laura Sticchi, Bianca Bruzzone, Alfredo Montecucco, Luca Pellegrini, Alessia Manca, Alexander Domnich, Angela Battistini, Bruno Kusznir Vitturi, Sonia Zacconi, Nicoletta Debarbieri, Giancarlo Icardi, Paolo Durando

**Affiliations:** 1 Department of Health Sciences, University of Genoa, 16132 Genoa, Italy; alborz.rahmani@edu.unige.it (A.R.); guglielmo.dini@unige.it (G.D.); andrea.orsi@unige.it (A.O.); laura.sticchi@unige.it (L.S.); alfredo.montecucco@edu.unige.it (A.M.); luca.pellegrini@edu.unige.it (L.P.); bruno.kusznir.vitturi@edu.unige.it (B.K.V.); sonia.zacconi@edu.unige.it (S.Z.); icardi@unige.it (G.I.); 2 Occupational Medicine Unit, IRCCS Ospedale Policlinico San Martino, 16132 Genoa, Italy; alessia.manca@hsanmartino.it (A.M.); nicoletta.debarbieri@hsanmartino.it (N.D.); 3 Hygiene Unit, IRCCS Ospedale Policlinico San Martino, 16132 Genoa, Italy; bianca.bruzzone@hsanmartino.it (B.B.); alexander.domnich@hsanmartino.it (A.D.); angela.battistini@hsanmartino.it (A.B.)

**Keywords:** COVID-19, mRNA vaccine, mass vaccination campaign, occupational health, healthcare workers, reactogenicity, adverse reactions

## Abstract

Vaccinations are a key prevention measure in fighting the COVID-19 pandemic. The BNT162b2 mRNA vaccine (BioNTech/Pfizer), the first to receive authorization, was widely used in the mass vaccination campaign in Italy. Healthcare workers were identified as a priority group for vaccination, but few studies have assessed its reactogenicity among the young working age population. An online survey was conducted to investigate the adverse reactions occurring in the 7 days following the first and second vaccination doses amongst resident doctors of the University of Genoa, employed at the IRCCS Ospedale Policlinico San Martino of Genoa, between 11 January and 16 March 2021. A total of 512 resident physicians were invited to participate in the study (female = 53.2%; mean age = 28.9 years), of whom 296 (female = 53.4%, mean age = 28.9 years) and 275 (female = 55.3%, mean age = 29.1 years) completed the survey after their first and second vaccination doses, respectively. In the 7 days following the first dose, most common adverse reactions were local pain (96.3%), fatigue (42.6%), headache (33.8%), arthromyalgia (28.0%), and 5.1% reported fever, while following the second dose, participants reported local pain (93.5%), fatigue (74.9%), headache (57.5%), arthromyalgia (58.2%), and fever (30.9%), with a higher prevalence among females. Systemic (but not local) reactions increased following the second vaccination, reaching severe intensity in 9.8% of participants and causing three or more events of moderate intensity in 23.7% of participants. Adverse reactions preventing regular daily activities could cause absenteeism among workers. These results can be useful to inform populations of young individuals, set expectations, and improve adherence to vaccination campaigns.

## 1. Introduction

At the end of 2019, the emergence of a novel coronavirus, named severe acute respiratory syndrome coronavirus 2 (SARS-CoV-2), caused the ongoing coronavirus disease 2019 (COVID-19) global pandemic. Soon after the genetic sequencing and isolation of this virus, researchers worldwide began studying and developing vaccine candidates. Indeed, vaccinations represent a key prevention measure in fighting infectious diseases, effectively reducing morbidity and mortality caused by a specific pathogen among a susceptible population. In December 2020, the first emergency use authorization for a vaccine for the prevention of COVID-19 was issued by the World Health Organization (WHO), the Food and Drug Administration (FDA), and the European Medicines Agency (EMA): a non-amplifying mRNA vaccine, called BNT162b2 [1,2,3]. This vaccine was also the first to receive full authorization for use by the FDA [4]. Messenger RNA vaccines provide the genetic code of the pathogen’s relevant antigen, in this case the spike protein of SARS-CoV-2, which is then translated by the host to form the relevant protein, which in turn induces specific humoral and cell-mediated immunity [5].

A strategic vaccination plan was set by the Ministry of Health, which put healthcare workers (HCWs) amongst the first prioritized categories to receive the vaccination [6]. Not only are these workers at an increased risk of exposure to the virus, potentially acquiring the infection and spreading the disease to patients and co-workers, but vaccinating individuals in this occupational category also increases the resilience of the healthcare system as a whole by preventing staff shortages [7]. Furthermore, according to Italian laws, vaccination of workers exposed to pathogens causing vaccine preventable diseases (VPDs) is considered a special measure of protection, to be implemented with other means of protection, such as personal and collective protective equipment [8]. Healthcare personnel in training, such as resident doctors, have been increasingly deployed on the front lines in the most critical phases of the pandemic, due in part to lack of staffing caused by infection among the workforce [9]. Indeed, previous studies have described the impact of SARS-CoV-2 infection due to occupational exposure on healthcare workers and resident doctors [10,11,12]. Extensive clinical trials were performed to evaluate the safety and efficacy of this vaccine [13], and further studies have assessed its real world reactogenicity and effectiveness [14,15]. Data from clinical trials of this vaccine have shown that participants reported local and systemic reactions, such as injection site pain, fatigue, and headache; greater reactogenicity was reported following the second dose and among the young adult age group [13]. Continued monitoring of reactogenicity of COVID-19 vaccines outside of clinical trial settings, particularly focusing on populations with an increased incidence of adverse reactions, may provide additional information for further implementation in the occupational settings (e.g., healthcare settings, schools, public institutions) and the community. Indeed, a thorough comprehension of the range of symptoms that vaccinations might cause is important both for the vaccinee, as well as for healthcare professionals who recommend and administer vaccines. Therefore, knowledge of the symptoms that could occur following immunization could be used, for healthcare professionals to prepare patients’ expectations, potentially leading to improved compliance and acceptance. Optimizing vaccine coverage is critical to the success of COVID-19 vaccination programs.

## 2. Materials and Methods

A cross-sectional observational study was performed using a self-administered electronic questionnaire, designed ex novo, and distributed with the open-source online software LimeSurvey (Version 4.3.28., LimeSurvey GmbH, Hamburg, Germany). The survey’s homepage reported an online informed consent form with specific information about the study purpose and the questionnaire’s general description. In addition to demographic questions (age, sex), the questionnaire contained various items dealing with different solicited local or systemic adverse events occurring in the seven days following the administration of the first and the second doses of the mRNA vaccine. The vaccination schedule consisted of a two-dose series of intramuscular injections in the deltoid muscle, of 0.3 mL of BNT162b2 vaccine (developed and produced by Pfizer/BioNTech, New York City, NY, USA/ Mainz, Germany) per dose, separated by 21 days, as recommended by the manufacturer and in accordance to national regulations. The study was carried out between 11 January and 16 March 2021. It involved the resident doctors of the University of Genoa employed at IRCCS Ospedale Policlinico San Martino of Genoa, Italy, the regional tertiary adult acute care reference hospital, which underwent COVID-19 immunization during the early phase of the national vaccination campaign. Vaccinated resident doctors were contacted by email and were invited to participate via a link to the survey, with a unique access token, on the same day of the vaccine administration. Participation was voluntary and every participant was required to agree with the LimeSurvey privacy policy. Participants were instructed to fill out the questionnaire at the end of each of the seven days after the vaccine administration. Three reminders were sent by email through the LimeSurvey software at different times during the week following the vaccination; on the 8th day, the survey ended, and data were exported.

The electronic form was structured in four sections: (1) demographic characteristics of the participants; (2) local adverse reactions; (3) systemic adverse reactions; and (4) rare adverse reactions (Supplementary material).

Most questions (local reactions: redness, swelling, pain; systemic reactions: fatigue, headache, joint/muscle pain, gastrointestinal symptoms) were array number types (7 × 5), in order to gather information on five different levels of symptom severity (absent, mild, moderate, severe, grade 4) for each of the seven days following the vaccination. Fever was assessed with array number types (7 × 6), based on recorded body temperatures (≥37.5 °C to 38.0 °C; >38.0 °C to 38.5 °C; >38.5 °C to 39.0 °C; >39.0 °C to 40.0 °C; >40.0 °C). Symptom severity definitions were based on (and adapted from) previously published documents [13,16], and are summarized in Table 1. The remaining questions (gender; chills; lymphadenomegaly; neurological symptoms; use of antipyretics/non-steroidal anti-inflammatory drugs—NSAIDs) were binary type and array type (7 × 2), with a yes/no answer for the 7 days of the study. When reported, rare adverse reactions (such as neurological symptoms) were investigated through a telephone interview by the Occupational Health Service (OHS), where participants were asked to thoroughly describe the event.

Partially completed surveys were discarded and only fully completed surveys (81 questions) were included in the final analysis. To account for the possible effects of the first dose administration on the second injection, differences between adverse reactions following each dose were evaluated using data from subjects that completed surveys after each dose of the vaccination.

All data were extracted by (and exported to) Microsoft Excel (Microsoft, Redmond, WA, USA) and then analyzed by Statistical Package for the Social Sciences (SPSS, IBM Corp., Armonk, NY, USA) software, version 22. Nominal and ordinal categorical variables were summarized and described as frequency and percentages. The Clopper–Pearson Exact method was used to calculate confidence intervals (CIs) for proportions. The McNemar test was used to compare paired proportions for non-parametric nominal data. The χ^2^ test and Fisher’s exact test were used for a univariate analysis of the association between sample characteristics and frequency of the reported adverse reactions. A two-tailed *p* < 0.05 was considered statistically significant.

Participants were informed that the results of the survey would be used for scientific purposes.

The study was managed by the Ethics Committee of the Liguria Region (administrative reference number: 631/2021 ID 11929). All activities were performed in compliance with the Declaration of Helsinki. Data were anonymized before the analysis. Personal information regarding all subjects included in the investigation was protected according to Italian law.

## 3. Results

A total of 512 resident doctors received COVID-19 vaccinations (female = 53.2%; mean age = 28.9 years SD 2.7) and were asked to complete diaries of their symptoms during the 7 days following vaccination. Among these, 365 participants entered the survey following the first administration (acceptance rate of 71.3%), as did 318 after the second vaccination (acceptance rate of 62.1%), of which 296 resident doctors fully completed the survey after the first dose administration (53.4% female, mean age of 28.9 years SD 2.6), and 275 completed the questionnaire after the second dose (55.3% female, mean age 29.1 years SD 2.9). Following the first dose administration, the most frequently reported local adverse reactions were redness (15.5%), swelling (29.4%), and pain (96.3%), while after the second dose administration, it was redness (17.1%), swelling (35.6%), and pain (93.5%). The complete description of local reactions is available in Table 2.

Concerning systemic reactions, most commonly reported adverse reactions following the first dose were fatigue (42.6%), headache (33.8%), muscle/joint pain (28.0%), lymph node enlargement (11.8%), and fever (5.1%), while after the second dose, these events were reported with a prevalence of fatigue (74.9%), headache (57.5%) muscle/joint pain (58.2%), lymphadenomegaly (18.2%), and fever (30.9%). The complete description of systemic reactions is available in Table 3.

Upon first dose administration, eight subjects reported neurological symptoms; after being contacted via phone call by the OHS medical team, six were excluded with reason: four cases of headaches who were already counted within the appropriate question, two cases of local and transient (<48 h) paresthesia affecting the vaccinated arm. Similarly, after the second vaccination dose, nine residents reported neurological symptoms, of whom three were excluded with reason: one case of a headache and two cases of local injection site paresthesia. Details of cases that were not excluded are reported in Table 4.

The majority of adverse reactions appeared in the first three days, subsiding within a median of three days from onset.

Median time of event onset for all local and systemic reactions was in the first day after both doses, with the exception of gastrointestinal symptoms occurring in the third day (IQR 2–5), and lymphadenomegaly in the second day following the first vaccination (IQR 1–4). Median time of event duration for all local and systemic reactions after both doses of vaccination ranged between 1 and 3 days, the longest being lymph node enlargement, lasting a median of 3 days (IQR 2–4) after each administration.

Following the administration of the first vaccine dose, participants reported a prevalence of antipyretics/NSAIDs use equal to 21.6% (95%CI 17.1–26.8), for a median duration of 1 day (IQR 1–2), while this proportion rose to 57.1% (95%CI 51.0–63.0), for a median duration of 2 days (IQR 1–2), after the second dose.

Subjects presenting at least one severe event that prevented regular daily activities were 11 after the first dose (3.7%) and 27 after the second dose (9.8%).

Excluding these severe cases, residents showing multiple moderate events that interfered with regular activities also increased following the second vaccination, as detailed in Table 5.

At the univariate analysis, upon the first vaccination dose, significant associations were found between being female and an increased reporting of headache (χ^2^ = 8.196, *p* = 0.004; OR = 2.06, 95%CI 1.25–3.38), and moderate pain (χ^2^ = 4.466, *p* = 0.035; OR = 1.79, 95%CI 1.10–2.90), while after the second dose it was positively associated with fatigue (χ^2^ = 3.988, *p* = 0.046; OR = 1.75, 95%CI 1.01–3.02), severe headache (χ^2^ = 4.752, *p* = 0.029; OR = 4.72, 95%CI 1.03–21.7), and muscle/joint pain (χ^2^ = 5.529, *p* = 0.019; OR = 1.79, 95%CI 1.10–2.90). Significant negative associations among age (per 1 year increase) and moderate fatigue (OR = 0.78, 95%CI 0.64–0.95), moderate headache (OR = 0.83, 95%CI 0.69–1.00), and lymph node enlargement (OR = 0.76, 95%CI 0.62–0.94) were found after the first vaccination; upon the second administration, age was positively associated only with redness of any intensity (OR = 1.10, 95%CI 1.01–1.21) and mild intensity (OR = 1.13, 95%CI 1.02–1.24).

When restricting data to the sample that completed the survey after each dose of the vaccination schedule (N = 200; female = 54.0%; mean age = 28.9 years SD 2.5), no significant difference was found concerning the local adverse reaction frequency between the first and second dose of the vaccination (with the exception of pain at the injection site—after the first dose = 97.0%, 95%CI 93.6–98.9; after the second dose = 92.5%, 95%CI 87.9–95.7; *p* = 0.05).

Regarding systemic adverse reactions, significant differences were present for most symptoms, with the exclusion of neurological symptoms (after the first dose = 0%, 95%CI 0.0–1.8; after the second dose = 2.0%, 95%CI 0.5–5.0; *p* = 0.13). Details are shown in Table 6.

## 4. Discussion and Conclusions

In this cross-sectional study, the first to the authors’ knowledge to investigate day-by-day differences in reactogenicity among young healthcare professionals, a high frequency of local and systemic adverse reactions was reported following immunization with the BNT162b2 mRNA COVID-19 vaccine. In particular, systemic reactions after the second dose were significantly more frequent compared to the first vaccination, in several instances preventing regular daily activities (around 1 every 10 doctors due to severe pain, fatigue, headache, and/or arthromyalgia), and with over half of the study population resorting to symptomatic treatment, such as fever lowering medications or NSAIDs. Furthermore, the combination of multiple moderate reactions that could interfere in daily activities also increased after the second vaccination, with almost one in four resident doctors showing three or more moderate reactions following the second dose. Indeed, when assessing the sample that had completed questionnaires after both vaccine administrations, we found that all systemic reactions, but not local ones, were significantly increased upon vaccine schedule completion. Our findings are in line with previously published data in phase 3 clinical trials [13], as well as in real-world effectiveness studies of COVID-19 mRNA vaccines [15,17,18]. Rare adverse reactions consisting of temporary neurological symptoms, such as paresthesia, vertigo, and confusional state were reported, as discussed in recent literature [19], although it is unclear if these were isolated manifestations, or caused or heightened by the concurrence of fever and other systemic reactions. Nevertheless, it is important to highlight that no hospitalization was reported and most adverse reactions occurred within the first 24 h after the vaccination and resolved completely within a median of 3 days. Finally, female resident doctors showed a higher frequency of headache, fatigue, and muscle and joint pain: an increase in systemic reactions among female healthcare workers was also shown in other recent investigations [20]. Previous data published by the Centers for Disease Control and Prevention (CDC) showed that 79.1% of adverse events reports after the first month of COVID-19 vaccination in the USA were made by women, whereas the female vaccinated population made up 61.2% [21]. Similarly, higher rates of adverse events among females have been reported following other immunizations, such as after seasonal influenza vaccination [22]. Differences in immune reactogenicity following vaccination between genders are considered to be caused by multiple factors, mainly biological factors, including hormonal [23], genetic [24,25], as well as perception and behavior [26,27].

The findings of the present study are strengthened by a high response rate of around 60%, considering the requirement of completion of a long and detailed survey with a 7-day data collection and without incentives for participants. Moreover, the sample population of trained medical professionals might increase the reliability of the data. These strengths might improve the generalizability of the results to other young working age groups, although the inclusion of a mostly homogenous population, particularly concerning age and occupational background, requires caution in applying these findings to all work settings. However, this study is limited in some aspects, due to the study design, with the possible introduction of non-response bias, recall bias, and self-report bias (e.g., preventive use of antipyretic analgesics or NSAIDs as prophylaxis of adverse reactions or as symptomatic treatment were not discernible, possibly causing an under-reporting of side effects. The description of the neurological symptoms was collected at the end of the survey, when complete resolution had already occurred in all reported cases).

Nonetheless, these findings could aid in informing specific populations of young individuals and, in turn, improving adherence to vaccination campaigns. From an occupational perspective, this is particularly relevant because the possibility of adverse reactions following vaccinations that prevent normal daily activities could cause absenteeism among workers. In this regard, workplace vaccination programs could consider these data when planning workforce population vaccinations. Occupational and public health physicians, through appropriate and informed pre- and post-vaccination counselling, could play a crucial role in setting expectations, particularly by emphasizing the benefit–risk ratio and positive framing of the mild and transient adverse effects, potentially alleviating anxiety in the post-vaccination period and, in turn, possibly reducing vaccine hesitancy [28]. The present study can aid in this endeavor, providing healthcare professionals and policy makers with up-to-date and real-world evidence from a working age population.

## Figures and Tables

**Table 1:** Classification of local and systemic adverse reaction severity following vaccination.

	Mild	Moderate	Severe	Grade 4
Local reactions				
**Redness**	>2.0 to 5.0 cm	>5.0 to 10.0 cm	>10.0 cm	Necrosis or exfoliative dermatitis
**Swelling**	>2.0 to 5.0 cm	>5.0 to 10.0 cm	>10.0 cm	Necrosis
**Pain**	Does not interfere with activity	Some interference with activity	Prevents daily activity	Emergency room visit or hospitalization for severe pain at the injection site.
**Systemic reactions**				
**Fatigue**	Does not interfere with activity	Some interference with activity	Prevents daily activity	Emergency room visit or hospitalization for severe fatigue.
**Headache**	Does not interfere with activity	Some interference with activity	Prevents daily activity	Emergency room visit or hospitalization for severe headache.
**Muscle/Joint pain**	Does not interfere with activity	Some interference with activity	Prevents daily activity	Emergency room visit or hospitalization for severe muscle pain or severe joint pain.
**Gastrointestinal symptoms**	Emesis 1 or 2 times and/or 2 or 3 loose stools in 24 h	Emesis > 2 times and/or 4 or 5 loose stools in 24 h	Requiring intravenous hydration and/or ≥6 loose stools in 24 h	Emergency room visit or hospitalization for severe vomiting and/or diarrhea.

**Table 2:** Reporting of local adverse reactions in the 7 days after the first and second vaccine dose administration, stratified by symptom severity.

	Dose 1	Dose 2
	N = 296	N = 275
Redness, *n* (% and 95%CI)		
Any	46 (15.5%, 95%CI 11.6–20.2)	47 (17.1%, 95%CI 12.8–22.1)
Mild	43 (14.5%, 95%CI 10.7–19.1)	39 (14.2%, 95%CI 10.3–18.9)
Moderate	3 (1.0%, 95%CI 0.2–2.9)	6 (2.2%, 95%CI 0.8–4.7)
Severe	0 (0)	2 (0.7%, 95%CI 0.1–2.6)
Grade 4	0 (0)	0 (0)
**Swelling, *n* (% and 95%CI)**		
Any	88 (29.7%, 95%CI 24.6–35.3)	98 (35.6%, 95%CI 30.0–41.6)
Mild	80 (27.0%, 95%CI 22.1–32.5)	90 (32.7%, 95%CI 27.2–38.6)
Moderate	8 (2.7%, 95%CI 1.2–5.3)	8 (2.9%, 95%CI 1.3–5.7)
Severe	0 (0)	0 (0)
Grade 4	0 (0)	0 (0)
**Pain at the injection site, *n* (% and 95%CI)**		
Any	285 (96.3%, 95%CI 93.4–98.1)	257 (93.5%, 95%CI 89.9–96.1)
Mild	133 (44.9%, 95%CI 39.2–50.8)	136 (49.5%, 95%CI 43.4–55.5)
Moderate	146 (49.3%, 95%CI 43.5–55.2)	116 (42.2%, 95%CI 36.3–48.3)
Severe	6 (2.0%, 95%CI 0.8–4.4)	5 (1.8%, 95%CI 0.6–4.2)
Grade 4	0 (0)	0 (0)

**Table 3:** Reporting of systemic adverse reactions in the 7 days after the first and second vaccine dose administration, stratified by symptom severity.

	Dose 1	Dose 2
	N = 296	N = 275
Fever, *n* (% and 95%CI)		
Any	15 (5.1%, 95%CI 2.9–8.2)	85 (30.9%, 95%CI 25.5–36.7)
≥37.5 °C to 38.0 °C	12 (4.1%, 95%CI 2.1–7.0)	58 (21.1%, 95%CI 16.4–26.4)
>38.0 °C to 38.5 °C	3 (1.0%, 95%CI 0.2–2.9)	18 (6.6%, 95%CI 3.9–10.2)
>38.5 °C to 39.0 °C	0 (0)	8 (2.9%, 95%CI 1.3–5.7)
>39.0 °C to 40.0 °C	0 (0)	1 (0.4%, 95%CI 0.0–2.0)
>40.0 °C	0 (0)	0 (0)
**Fatigue, *n* (% and 95%CI)**		
Any	126 (42.6%, 95%CI 36.9–48.4)	206 (74.9%, 95%CI 69.4–79.9)
Mild	85 (28.7%, 95%CI 23.6–34.2)	82 (29.8%, 95%CI 24.5–35.6)
Moderate	41 (13.9%, 95%CI 10.1–18.3)	107 (38.9%, 95%CI 33.1–45.0)
Severe	0 (0)	17 (6.2%, 95%CI 3.6–9.7)
Grade 4	0 (0)	0 (0)
**Headache, *n* (% and 95%CI)**		
Any	100 (33.8%, 95%CI 28.4–39.5)	158 (57.5%, 95%CI 51.4–63.4)
Mild	56 (18.9%, 95%CI 14.6–23.9)	77 (28.0%, 95%CI 22.8–33.7)
Moderate	40 (13.5%, 95%CI 9.8–17.9)	68 (24.7%, 95%CI 19.7–30.3)
Severe	4 (1.4%, 95%CI 0.4–3.4)	13 (4.7%, 95%CI 2.5–8.0)
Grade 4	0 (0)	0 (0)
**Gastrointestinal symptoms, *n* (% and 95%CI)**		
Any	17 (5.7%, 95%CI 3.4–9.0)	40 (14.5%, 95%CI 10.6–19.3)
Mild	17 (5.7%, 95%CI 3.4–9.0)	37 (13.5%, 95%CI 9.7–18.1)
Moderate	0 (0)	3 (1.1%, 95%CI 0.2–3.2)
Severe	0 (0)	0 (0)
Grade 4	0 (0)	0 (0)
**Muscle/Joint pain, *n* (% and 95%CI)**		
Any	83 (28.0%, 95%CI 23.0–33.5)	160 (58.2%, 95%CI 52.1–64.1)
Mild	56 (18.9%, 95%CI 14.6–23.9)	72 (26.2%, 95%CI 21.1–31.8)
Moderate	25 (8.5%, 95%CI 5.5–12.2)	77 (28.0%, 95%CI 22.8–33.7)
Severe	2 (0.7%, 95%CI 0.1–2.4)	11 (4.0%, 95%CI 2.0–7.0)
Grade 4	0 (0)	0 (0)
**Chills**, ***n* (% and 95%CI)**		
Any	35 (11.8%, 95%CI 8.4–16.1)	105 (38.2%, 95%CI 32.4–44.2)
**Lymph node enlargement, *n* (% and 95%CI)**		
Any	35 (11.8%, 95%CI 8.4–16.1)	50 (18.2%, 95%CI 13.8–23.3)
**Neurological symptoms, *n* (% and 95%CI)**		
Any	2 (0.7%, 95%CI 0.1–2.4)	6 (2.2%, 95%CI 0.8–4.7)

**Table 4:** Narrative description of cases reporting neurological symptoms included in the study.

Dose 1	Dose 2
- Male, 30 years old, reported vertigo in the 24 h following vaccination	- Female, 28 years old, reported bilateral paresthesia of the arms and hands, lasting 24 h
- Female, 29 years old, reported paresthesia in the left thigh for 4 days following vaccination	- Male, 28 years old, reported vertigo in the 24 h following vaccination
	- Female, 27 years old, reported vertigo in the 24 h following vaccination
	- Female, 31 years old, reported nervousness and confusion in the 24 h following vaccination
	- Female, 29 years old, reported visual hallucinations with concurrent fever, the night after vaccination
	- Female, 25 years old, reported sensory hypersensitivity in the 48 h following the vaccination

**Table 5:** Prevalence of reporting of one of more moderate adverse reactions interfering with activities following the first and second vaccination doses, among subjects without severe reactions.

No. of Moderate Reactions	Dose 1 (285) *n* (%)	Dose 2 (248) *n* (%)
1	112 (39.3)	61 (24.6)
2	39 (13.7)	40 (16.1)
3	14 (4.9)	44 (17.7)
4	2 (0.7)	15 (6.0)

**Table 6:** Difference in reporting of systemic adverse reactions in the 7 days after the first and second vaccine dose administration.

	Dose 1	Dose 2	Sig.
Fever	5.5% (95%CI 2.8–9.6)	32.0% (95%CI 25.6–38.9)	0.000
Fatigue	41.5% (95%CI 34.6–48.7)	74.5% (95%CI 67.9–80.4)	0.000
Chills	12.0% (95%CI 7.8–17.3)	40.0% (95%CI 33.2–47.1)	0.000
Headache	34.5% (95%CI 27.9–41.5)	55.5% (95%CI 48.3–62.5)	0.000
Muscle/joint pain	25.5% (95%CI 19.6–32.1)	56.0% (95%CI 48.8–63.0)	0.000
Gastrointestinal symptoms	6.0% (95%CI 3.1–10.2)	15.0% (95%CI 10.4–20.7)	0.003
Lymph node enlargement	10.5% (95%CI 6.6–15.6)	18.5% (95%CI 13.4–24.6)	0.015

## Data Availability

The data that support the findings of this study are available on request from the corresponding author. The data are not publicly available due to privacy restrictions.

## Supplementary Materials

The following are available online at https://www.mdpi.com/article/10.3390/vaccines9111269/s1, the Italian electronic form exported from LimeSurvey in a printable format and the English transcript.
