# Supplementary material for: Reactogenicity of BNT162b2 mRNA COVID-19 Vaccine in a Young Working Age Population: A Survey among Medical School Residents, within a Mass Vaccination Campaign, in a Regional Reference Teaching Hospital in Italy
Source: Vaccines (Basel). 2021 Nov 3;9(11):1269. doi: 10.3390/vaccines9111269 (PMC8625320; doi:10.3390/vaccines9111269)
Supplement: Supplementary file 1 [file vaccines-09-01269-s001.zip › Survey/limesurvey.EN.def.pdf]

**This questionnaire is designed to investigate local and systemic reactions following the COVID-19 mRNA vaccination. The participant is requested to carefully complete the questionnaire within 7 days after the vaccination. These data are very important to research during the vaccination campaign in Italy. Please fill in the questionnaire daily, and select only one answer for each day. The questionnaire can be saved, resumed and modified until it is submitted by the participant or until the deadline.**
**Section A: Section 1 – Demographics**
A1. First and last name:
A2. Gender: Female/Male/Non-binary
A3. Age: years
**Section B: Section 2 - COVID-19 mRNA Vaccine: LOCAL EFFECTS**
B1. Did you have redness at the injection site? Day 1: none / mild, >2.0 to 5.0 cm / moderate, >5.0 to 10.0 cm / severe, >10.0 cm / Grade4, Necrosis or exfoliative dermatitis
B2. Did you have redness at the injection site? Day 2: none / mild, >2.0 to 5.0 cm / moderate, >5.0 to 10.0 cm / severe, >10.0 cm / Grade4, Necrosis or exfoliative dermatitis
B3. Did you have redness at the injection site? Day 3: none / mild, >2.0 to 5.0 cm / moderate, >5.0 to 10.0 cm / severe, >10.0 cm / Grade4, Necrosis or exfoliative dermatitis
B4. Did you have redness at the injection site? Day 4: none / mild, >2.0 to 5.0 cm / moderate, >5.0 to 10.0 cm / severe, >10.0 cm / Grade4, Necrosis or exfoliative dermatitis
B5. Did you have redness at the injection site? Day 5: none / mild, >2.0 to 5.0 cm / moderate, >5.0 to 10.0 cm / severe, >10.0 cm / Grade4, Necrosis or exfoliative dermatitis
B6. Did you have redness at the injection site? Day 6: none / mild, >2.0 to 5.0 cm / moderate, >5.0 to 10.0 cm / severe, >10.0 cm / Grade4, Necrosis or exfoliative dermatitis
B7. Did you have redness at the injection site? Day 7: none / mild, >2.0 to 5.0 cm / moderate, >5.0 to 10.0 cm / severe, >10.0 cm / Grade4, Necrosis or exfoliative dermatitis
B8. Did you have swelling at the injection site? Day 1: none / mild, >2.0 to 5.0 cm / moderate, >5.0 to 10.0 cm / severe, >10.0 cm / Grade4, Necrosis
B9. Did you have swelling at the injection site? Day 2: none / mild, >2.0 to 5.0 cm / moderate, >5.0 to 10.0 cm / severe, >10.0 cm / Grade4, Necrosis
B10. Did you have swelling at the injection site? Day 3: none / mild, >2.0 to 5.0 cm / moderate, >5.0 to 10.0 cm / severe, >10.0 cm / Grade4, Necrosis
B11. Did you have swelling at the injection site? Day 4: none / mild, >2.0 to 5.0 cm / moderate, >5.0 to 10.0 cm / severe, >10.0 cm / Grade4, Necrosis
B12. Did you have swelling at the injection site? Day 5: none / mild, >2.0 to 5.0 cm / moderate, >5.0 to 10.0 cm / severe, >10.0 cm / Grade4, Necrosis
B13. Did you have swelling at the injection site? Day 6: none / mild, >2.0 to 5.0 cm / moderate, >5.0 to 10.0 cm / severe, >10.0 cm / Grade4, Necrosis
B14. Did you have swelling at the injection site? Day 7: none / mild, >2.0 to 5.0 cm / moderate, >5.0 to 10.0 cm / severe, >10.0 cm / Grade4, Necrosis
B15. Did you have pain at the injection site? Day 1: none / mild, does not interfere with activity / moderate, some interference with activity / severe, prevents daily activity / Grade4, Emergency room visit or hospitalization for severe pain at the injection site
B16. Did you have pain at the injection site? Day 2: none / mild, does not interfere with activity / moderate, some interference with activity / severe, prevents daily activity / Grade4, Emergency room visit or hospitalization for severe pain at the injection site

B17. Did you have pain at the injection site? Day 3: none / mild, does not interfere with activity / moderate, some interference with activity / severe, prevents daily activity / Grade4, Emergency room visit or hospitalization for severe pain at the injection site
B18. Did you have pain at the injection site? Day 4: none / mild, does not interfere with activity / moderate, some interference with activity / severe, prevents daily activity / Grade4, Emergency room visit or hospitalization for severe pain at the injection site
B19. Did you have pain at the injection site? Day 5: none / mild, does not interfere with activity / moderate, some interference with activity / severe, prevents daily activity / Grade4, Emergency room visit or hospitalization for severe pain at the injection site
B20. Did you have pain at the injection site? Day 6: none / mild, does not interfere with activity / moderate, some interference with activity / severe, prevents daily activity / Grade4, Emergency room visit or hospitalization for severe pain at the injection site
B21. Did you have pain at the injection site? Day 7: none / mild, does not interfere with activity / moderate, some interference with activity / severe, prevents daily activity / Grade4, Emergency room visit or hospitalization for severe pain at the injection site
**Section C: Section 2 - COVID-19 mRNA Vaccine: SYSTEMIC EFFECTS**
C1. Did you have a fever? Day 1: no / ≥37.5°C to 38.0°C / >38.0°C to 38.5°C / >38.5°C to 39.0°C / >39.0°C to 40.0°C / >40.0°C
C2. Did you have a fever? Day 2: no / ≥37.5°C to 38.0°C / >38.0°C to 38.5°C / >38.5°C to 39.0°C / >39.0°C to 40.0°C / >40.0°C
C3. Did you have a fever? Day 3: no / ≥37.5°C to 38.0°C / >38.0°C to 38.5°C / >38.5°C to 39.0°C / >39.0°C to 40.0°C / >40.0°C
C4. Did you have a fever? Day 4: no / ≥37.5°C to 38.0°C / >38.0°C to 38.5°C / >38.5°C to 39.0°C / >39.0°C to 40.0°C / >40.0°C
C5. Did you have a fever? Day 5: no / ≥37.5°C to 38.0°C / >38.0°C to 38.5°C / >38.5°C to 39.0°C / >39.0°C to 40.0°C / >40.0°C
C6. Did you have a fever? Day 6: no / ≥37.5°C to 38.0°C / >38.0°C to 38.5°C / >38.5°C to 39.0°C / >39.0°C to 40.0°C / >40.0°C
C7. Did you have a fever? Day 7: no / ≥37.5°C to 38.0°C / >38.0°C to 38.5°C / >38.5°C to 39.0°C / >39.0°C to 40.0°C / >40.0°C
C8. Did you experience fatigue? Day 1: none / mild, does not interfere with activity / moderate, some interference with activity / severe, prevents daily activity / Grade4, Emergency room visit or hospitalization for severe fatigue
C9. Did you experience fatigue? Day 2: none / mild, does not interfere with activity / moderate, some interference with activity / severe, prevents daily activity / Grade4, Emergency room visit or hospitalization for severe fatigue
C10. Did you experience fatigue? Day 3: none / mild, does not interfere with activity / moderate, some interference with activity / severe, prevents daily activity / Grade4, Emergency room visit or hospitalization for severe fatigue
C11. Did you experience fatigue? Day 4: none / mild, does not interfere with activity / moderate, some interference with activity / severe, prevents daily activity / Grade4, Emergency room visit or hospitalization for severe fatigue
C12. Did you experience fatigue? Day 5: none / mild, does not interfere with activity / moderate, some interference with activity / severe, prevents daily activity / Grade4, Emergency room visit or hospitalization for severe fatigue
C13. Did you experience fatigue? Day 6: none / mild, does not interfere with activity / moderate, some interference with activity / severe, prevents daily activity / Grade4, Emergency room visit or hospitalization for severe fatigue

C14. Did you experience fatigue? Day 7: none / mild, does not interfere with activity / moderate, some interference with activity / severe, prevents daily activity / Grade4, Emergency room visit or hospitalization for severe fatigue
C15. Did you have chills? Day 1: no / yes
C16. Did you have chills? Day 2: no / yes
C17. Did you have chills? Day 3: no / yes
C18. Did you have chills? Day 4: no / yes
C19. Did you have chills? Day 5: no / yes
C20. Did you have chills? Day 6: no / yes
C21. Did you have chills? Day 7: no / yes
C22. Have you had a headache? Day 1: none / mild, does not interfere with activity / moderate, some interference with activity / severe, prevents daily activity / Grade4, Emergency room visit or hospitalization for severe headache
C23. Have you had a headache? Day 2: none / mild, does not interfere with activity / moderate, some interference with activity / severe, prevents daily activity / Grade4, Emergency room visit or hospitalization for severe headache
C24. Have you had a headache? Day 3: none / mild, does not interfere with activity / moderate, some interference with activity / severe, prevents daily activity / Grade4, Emergency room visit or hospitalization for severe headache
C25. Have you had a headache? Day 4: none / mild, does not interfere with activity / moderate, some interference with activity / severe, prevents daily activity / Grade4, Emergency room visit or hospitalization for severe headache
C26. Have you had a headache? Day 5: none / mild, does not interfere with activity / moderate, some interference with activity / severe, prevents daily activity / Grade4, Emergency room visit or hospitalization for severe headache
C27. Have you had a headache? Day 6: none / mild, does not interfere with activity / moderate, some interference with activity / severe, prevents daily activity / Grade4, Emergency room visit or hospitalization for severe headache
C28. Have you had a headache? Day 7: none / mild, does not interfere with activity / moderate, some interference with activity / severe, prevents daily activity / Grade4, Emergency room visit or hospitalization for severe headache
C29. Have you had any muscle/joint pain? Day 1: none / mild, does not interfere with activity / moderate, some interference with activity / severe, prevents daily activity / Grade4, Emergency room visit or hospitalization for severe muscle/joint pain
C30. Have you had any muscle/joint pain? Day 2: none / mild, does not interfere with activity / moderate, some interference with activity / severe, prevents daily activity / Grade4, Emergency room visit or hospitalization for severe muscle/joint pain
C31. Have you had any muscle/joint pain? Day 3: none / mild, does not interfere with activity / moderate, some interference with activity / severe, prevents daily activity / Grade4, Emergency room visit or hospitalization for severe muscle/joint pain
C32. Have you had any muscle/joint pain? Day 4: none / mild, does not interfere with activity / moderate, some interference with activity / severe, prevents daily activity / Grade4, Emergency room visit or hospitalization for severe muscle/joint pain
C33. Have you had any muscle/joint pain? Day 5: none / mild, does not interfere with activity / moderate, some interference with activity / severe, prevents daily activity / Grade4, Emergency room visit or hospitalization for severe muscle/joint pain
C34. Have you had any muscle/joint pain? Day 6: none / mild, does not interfere with activity / moderate, some interference with activity / severe, prevents daily activity / Grade4, Emergency room visit or hospitalization for severe muscle/joint pain

C35. Have you had any muscle/joint pain? Day 7: none / mild, does not interfere with activity / moderate, some interference with activity / severe, prevents daily activity / Grade4, Emergency room visit or hospitalization for severe muscle/joint pain
C36. Have you had any gastrointestinal symptoms? Day 1: none / mild, emesis 1 or 2 times and-or 2 or 3 loose stools in 24 hours / moderate, emesis >2 times and-or 4 or 5 loose stools in 24 hours / severe, requiring intravenous hydration and-or ≥6 loose stools in 24 hours / Grade4, emergency room visit or hospitalization for severe vomiting and/or diarrhea
C37. Have you had any gastrointestinal symptoms? Day 2: none / mild, emesis 1 or 2 times and-or 2 or 3 loose stools in 24 hours / moderate, emesis >2 times and-or 4 or 5 loose stools in 24 hours / severe, requiring intravenous hydration and-or ≥6 loose stools in 24 hours / Grade4, emergency room visit or hospitalization for severe vomiting and/or diarrhea
C38. Have you had any gastrointestinal symptoms? Day 3: none / mild, emesis 1 or 2 times and-or 2 or 3 loose stools in 24 hours / moderate, emesis >2 times and-or 4 or 5 loose stools in 24 hours / severe, requiring intravenous hydration and-or ≥6 loose stools in 24 hours / Grade4, emergency room visit or hospitalization for severe vomiting and/or diarrhea
C39. Have you had any gastrointestinal symptoms? Day 4: none / mild, emesis 1 or 2 times and-or 2 or 3 loose stools in 24 hours / moderate, emesis >2 times and-or 4 or 5 loose stools in 24 hours / severe, requiring intravenous hydration and-or ≥6 loose stools in 24 hours / Grade4, emergency room visit or hospitalization for severe vomiting and/or diarrhea
C40. Have you had any gastrointestinal symptoms? Day 5: none / mild, emesis 1 or 2 times and-or 2 or 3 loose stools in 24 hours / moderate, emesis >2 times and-or 4 or 5 loose stools in 24 hours / severe, requiring intravenous hydration and-or ≥6 loose stools in 24 hours / Grade4, emergency room visit or hospitalization for severe vomiting and/or diarrhea
C41. Have you had any gastrointestinal symptoms? Day 6: none / mild, emesis 1 or 2 times and-or 2 or 3 loose stools in 24 hours / moderate, emesis >2 times and-or 4 or 5 loose stools in 24 hours / severe, requiring intravenous hydration and-or ≥6 loose stools in 24 hours / Grade4, emergency room visit or hospitalization for severe vomiting and/or diarrhea
C42. Have you had any gastrointestinal symptoms? Day 7: none / mild, emesis 1 or 2 times and-or 2 or 3 loose stools in 24 hours / moderate, emesis >2 times and-or 4 or 5 loose stools in 24 hours / severe, requiring intravenous hydration and-or ≥6 loose stools in 24 hours / Grade4, emergency room visit or hospitalization for severe vomiting and/or diarrhea
C43. Did you use antipyretics / NSAIDs? Day 1: no / yes
C44. Did you use antipyretics / NSAIDs? Day 2: no / yes
C45. Did you use antipyretics / NSAIDs? Day 3: no / yes
C46. Did you use antipyretics / NSAIDs? Day 4: no / yes
C47. Did you use antipyretics / NSAIDs? Day 5: no / yes
C48. Did you use antipyretics / NSAIDs? Day 6: no / yes
C49. Did you use antipyretics / NSAIDs? Day 7: no / yes
**Section D: Section 2 - COVID-19 mRNA Vaccine: RARE ADVERSE EVENTS**
D1. Did you have any enlargement of the lymph nodes? Day 1: no / yes
D2. Did you have any enlargement of the lymph nodes? Day 2: no / yes
D3. Did you have any enlargement of the lymph nodes? Day 3: no / yes
D4. Did you have any enlargement of the lymph nodes? Day 4: no / yes
D5. Did you have any enlargement of the lymph nodes? Day 5: no / yes
D6. Did you have any enlargement of the lymph nodes? Day 6: no / yes
D7. Did you have any enlargement of the lymph nodes? Day 7: no / yes
D8. Have you had any symptoms affecting your nervous system? Day 1: no / yes
D9. Have you had any symptoms affecting your nervous system? Day 2: no / yes

D10. Have you had any symptoms affecting your nervous system? Day 3: no / yes
D11. Have you had any symptoms affecting your nervous system? Day 4: no / yes
D12. Have you had any symptoms affecting your nervous system? Day 5: no / yes
D13. Have you had any symptoms affecting your nervous system? Day 6: no / yes
D14. Have you had any symptoms affecting your nervous system? Day 7: no / yes
IMPORTANT: In the event of reactions occurring after 7 days from vaccination and/or clinical manifestations not included among those previously reported, please contact the Health Surveillance Service of the University.<br><br>The Health Surveillance Service - Occupational Medicine Unit, and the Hygiene Unit of the University of Genova remain at your complete disposal, for any information and counselling.<br><br>Contacts<br>...<br><br>Thank you for participating in this survey.
