# Supplementary material for: Reactogenicity of BNT162b2 mRNA COVID-19 Vaccine in a Young Working Age Population: A Survey among Medical School Residents, within a Mass Vaccination Campaign, in a Regional Reference Teaching Hospital in Italy
Source: Vaccines (Basel). 2021 Nov 3;9(11):1269. doi: 10.3390/vaccines9111269 (PMC8625320; doi:10.3390/vaccines9111269)
Supplement: Supplementary file 1 [file vaccines-09-01269-s001.zip › Survey/limesurvey.pdf]

LimeSurvey

Questo questionario è finalizzato a indagare le reazioni locali e sistemiche della vaccinazione mRNA COVID-19. Si prega il partecipante di compilare con attenzione il questionario nei 7 giorni a partire da quello della vaccinazione. Questi dati sono molto importanti in ambito di ricerca applicata durante la campagna vaccinale in Italia.

Si prega di compilare il questionario giornalmente, e di selezionare una sola risposta per ogni giorno. Il questionario può essere salvato, ripreso e modificato fino all'invio da parte del partecipante o alla scadenza prevista.

Sezione A: Sezione 1 - Anagrafica

A1. **Nome e Cognome**

A2. **Genere**

Femminile ☐
Maschile ☐
Non-binario ☐

A3. **Età**

Sezione B: Sezione 2 - Vaccino COVID-19 mRNA: EFFETTI LOCALI

B1.

Hai avuto arrossamento in sede di iniezione?

Giorno 1

Lieve: >2,0 a 5,0 cm; moderato: >5,0 a 10,0 cm; grave: >10,0 cm; grado 4: necrosi o dermatite esfoliativa

Grado 4 ☐
Grave ☐
Moderato ☐

Lieve ☐

Nessuno ☐

B2.

Hai avuto arrossamento in sede di iniezione?

Giorno 2

Lieve: >2,0 a 5,0 cm; moderato: >5,0 a 10,0 cm; grave: >10,0 cm; grado 4: necrosi o dermatite esfoliativa

Grado 4 ☐

Grave ☐

Moderato ☐

Lieve ☐

Nessuno ☐

B3.

Hai avuto arrossamento in sede di iniezione?

Giorno 3

Lieve: >2,0 a 5,0 cm; moderato: >5,0 a 10,0 cm; grave: >10,0 cm; grado 4: necrosi o dermatite esfoliativa

Grado 4 ☐

Grave ☐

Moderato ☐

Lieve ☐

Nessuno ☐

B4.

Hai avuto arrossamento in sede di iniezione?

Giorno 4

Lieve: >2,0 a 5,0 cm; moderato: >5,0 a 10,0 cm; grave: >10,0 cm; grado 4: necrosi o dermatite esfoliativa

Grado 4 ☐

Grave ☐

Moderato ☐

Lieve ☐

Nessuno ☐

LimeSurvey

B5.

Hai avuto arrossamento in sede di iniezione?

Giorno 5

Lieve: >2,0 a 5,0 cm; moderato: >5,0 a 10,0 cm; grave: >10,0 cm; grado 4: necrosi o dermatite esfoliativa

- Grado 4 ☐
- Grave ☐
- Moderato ☐
- Lieve ☐
- Nessuno ☐

B6.

Hai avuto arrossamento in sede di iniezione?

Giorno 6

Lieve: >2,0 a 5,0 cm; moderato: >5,0 a 10,0 cm; grave: >10,0 cm; grado 4: necrosi o dermatite esfoliativa

- Grado 4 ☐
- Grave ☐
- Moderato ☐
- Lieve ☐
- Nessuno ☐

B7.

Hai avuto arrossamento in sede di iniezione?

Giorno 7

Lieve: >2,0 a 5,0 cm; moderato: >5,0 a 10,0 cm; grave: >10,0 cm; grado 4: necrosi o dermatite esfoliativa

- Grado 4 ☐
- Grave ☐
- Moderato ☐
- Lieve ☐
- Nessuno ☐

LimeSurvey

B8.

Hai avuto gonfiore in sede di iniezione?

Giorno 1

Lieve: >2,0 a 5,0 cm; moderato: >5,0 a 10,0 cm; grave: >10,0 cm; grado 4: necrosi

- Grado 4 ☐
- Grave ☐
- Moderato ☐
- Lieve ☐
- Nessuno ☐

B9.

Hai avuto gonfiore in sede di iniezione?

Giorno 2

Lieve: >2,0 a 5,0 cm; moderato: >5,0 a 10,0 cm; grave: >10,0 cm; grado 4: necrosi

- Grado 4 ☐
- Grave ☐
- Moderato ☐
- Lieve ☐
- Nessuno ☐

B10.

Hai avuto gonfiore in sede di iniezione?

Giorno 3

Lieve: >2,0 a 5,0 cm; moderato: >5,0 a 10,0 cm; grave: >10,0 cm; grado 4: necrosi

- Grado 4 ☐
- Grave ☐
- Moderato ☐
- Lieve ☐
- Nessuno ☐

LimeSurvey

B11.

Hai avuto gonfiore in sede di iniezione?

Giorno 4

Lieve: >2,0 a 5,0 cm; moderato: >5,0 a 10,0 cm; grave: >10,0 cm; grado 4: necrosi

Grado 4 ☐

Grave ☐

Moderato ☐

Lieve ☐

Nessuno ☐

B12.

Hai avuto gonfiore in sede di iniezione?

Giorno 5

Lieve: >2,0 a 5,0 cm; moderato: >5,0 a 10,0 cm; grave: >10,0 cm; grado 4: necrosi

Grado 4 ☐

Grave ☐

Moderato ☐

Lieve ☐

Nessuno ☐

B13.

Hai avuto gonfiore in sede di iniezione?

Giorno 6

Lieve: >2,0 a 5,0 cm; moderato: >5,0 a 10,0 cm; grave: >10,0 cm; grado 4: necrosi

Grado 4 ☐

Grave ☐

Moderato ☐

Lieve ☐

Nessuno ☐

LimeSurvey

B14.

Hai avuto gonfiore in sede di iniezione?

Giorno 7

Lieve: >2,0 a 5,0 cm; moderato: >5,0 a 10,0 cm; grave: >10,0 cm; grado 4: necrosi

- Grado 4 ☐
- Grave ☐
- Moderato ☐
- Lieve ☐
- Nessuno ☐

B15.

Hai avuto dolore in sede di iniezione?

Giorno 1

Lieve: non interferisce con l'attività; moderato: interferisce con l'attività; grave: impedisce l'attività quotidiana; grado 4: visita al pronto soccorso o ricovero in ospedale per forte dolore al sito di iniezione.

- Grado 4 ☐
- Grave ☐
- Moderato ☐
- Lieve ☐
- Nessuno ☐

B16.

Hai avuto dolore in sede di iniezione?

Giorno 2

Lieve: non interferisce con l'attività; moderato: interferisce con l'attività; grave: impedisce l'attività quotidiana; grado 4: visita al pronto soccorso o ricovero in ospedale per forte dolore al sito di iniezione.

- Grado 4 ☐
- Grave ☐
- Moderato ☐
- Lieve ☐
- Nessuno ☐

B17.

Hai avuto dolore in sede di iniezione?

Giorno 3

Lieve: non interferisce con l'attività; moderato: interferisce con l'attività; grave: impedisce l'attività quotidiana; grado 4: visita al pronto soccorso o ricovero in ospedale per forte dolore al sito di iniezione.

- Grado 4 ☐
- Grave ☐
- Moderato ☐
- Lieve ☐
- Nessuno ☐

B18.

Hai avuto dolore in sede di iniezione?

Giorno 4

Lieve: non interferisce con l'attività; moderato: interferisce con l'attività; grave: impedisce l'attività quotidiana; grado 4: visita al pronto soccorso o ricovero in ospedale per forte dolore al sito di iniezione.

- Grado 4 ☐
- Grave ☐
- Moderato ☐
- Lieve ☐
- Nessuno ☐

B19.

Hai avuto dolore in sede di iniezione?

Giorno 5

Lieve: non interferisce con l'attività; moderato: interferisce con l'attività; grave: impedisce l'attività quotidiana; grado 4: visita al pronto soccorso o ricovero in ospedale per forte dolore al sito di iniezione.

- Grado 4 ☐
- Grave ☐
- Moderato ☐
- Lieve ☐
- Nessuno ☐

B20.

Hai avuto dolore in sede di iniezione?

Giorno 6

Lieve: non interferisce con l'attività; moderato: interferisce con l'attività; grave: impedisce l'attività quotidiana; grado 4: visita al pronto soccorso o ricovero in ospedale per forte dolore al sito di iniezione.

- Grado 4 ☐
- Grave ☐
- Moderato ☐
- Lieve ☐
- Nessuno ☐

B21.

Hai avuto dolore in sede di iniezione?

Giorno 7

Lieve: non interferisce con l'attività; moderato: interferisce con l'attività; grave: impedisce l'attività quotidiana; grado 4: visita al pronto soccorso o ricovero in ospedale per forte dolore al sito di iniezione.

- Grado 4 ☐
- Grave ☐
- Moderato ☐
- Lieve ☐
- Nessuno ☐

Sezione C: Sezione 2 - Vaccino COVID-19 mRNA: EFFETTI SISTEMICI

C1.

Hai avuto febbre?

Giorno 1

- >40,0°C ☐
- >39,0°C a 40,0°C ☐
- >38,5°C a 39,0°C ☐
- >38,0°C a 38,5°C ☐
- ≥37,5°C a 38,0°C ☐
- No ☐

LimeSurvey

C2.

Hai avuto febbre?

Giorno 2

- >40,0°C ☐
- >39,0°C a 40,0°C ☐
- >38,5°C a 39,0°C ☐
- >38,0°C a 38,5°C ☐
- ≥37,5°C a 38,0°C ☐
- No ☐

C3.

Hai avuto febbre?

Giorno 3

- >40,0°C ☐
- >39,0°C a 40,0°C ☐
- >38,5°C a 39,0°C ☐
- >38,0°C a 38,5°C ☐
- ≥37,5°C a 38,0°C ☐
- No ☐

C4.

Hai avuto febbre?

Giorno 4

- >40,0°C ☐
- >39,0°C a 40,0°C ☐
- >38,5°C a 39,0°C ☐
- >38,0°C a 38,5°C ☐
- ≥37,5°C a 38,0°C ☐
- No ☐

C5.

Hai avuto febbre?

Giorno 5

- >40,0°C ☐
- >39,0°C a 40,0°C ☐
- >38,5°C a 39,0°C ☐
- >38,0°C a 38,5°C ☐
- ≥37,5°C a 38,0°C ☐
- No ☐

C6.

Hai avuto febbre?

Giorno 6

- >40,0°C ☐
- >39,0°C a 40,0°C ☐
- >38,5°C a 39,0°C ☐
- >38,0°C a 38,5°C ☐
- ≥37,5°C a 38,0°C ☐
- No ☐

C7.

Hai avuto febbre?

Giorno 7

- >40,0°C ☐
- >39,0°C a 40,0°C ☐
- >38,5°C a 39,0°C ☐
- >38,0°C a 38,5°C ☐
- ≥37,5°C a 38,0°C ☐
- No ☐

C8.

Hai avuto astenia?

Giorno 1

Lieve: non interferisce con l'attività; moderato: una limitata interferenza con l'attività; grave: impedisce l'attività quotidiana; grado 4: visita al pronto soccorso o ricovero in ospedale per grave affaticamento

- Nessuno ☐
- Lieve ☐
- Moderato ☐
- Grave ☐
- Grado 4 ☐

C9.

Hai avuto astenia?

Giorno 2

Lieve: non interferisce con l'attività; moderato: una limitata interferenza con l'attività; grave: impedisce l'attività quotidiana; grado 4: visita al pronto soccorso o ricovero in ospedale per grave affaticamento

- Nessuno ☐
- Lieve ☐
- Moderato ☐
- Grave ☐
- Grado 4 ☐

C10.

Hai avuto astenia?

Giorno 3

Lieve: non interferisce con l'attività; moderato: una limitata interferenza con l'attività; grave: impedisce l'attività quotidiana; grado 4: visita al pronto soccorso o ricovero in ospedale per grave affaticamento

- Nessuno ☐
- Lieve ☐
- Moderato ☐
- Grave ☐
- Grado 4 ☐

C11.

Hai avuto astenia?

Giorno 4

Lieve: non interferisce con l'attività; moderato: una limitata interferenza con l'attività; grave: impedisce l'attività quotidiana; grado 4: visita al pronto soccorso o ricovero in ospedale per grave affaticamento

- Nessuno ☐
- Lieve ☐
- Moderato ☐
- Grave ☐
- Grado 4 ☐

C12.

Hai avuto astenia?

Giorno 5

Lieve: non interferisce con l'attività; moderato: una limitata interferenza con l'attività; grave: impedisce l'attività quotidiana; grado 4: visita al pronto soccorso o ricovero in ospedale per grave affaticamento

- Nessuno ☐
- Lieve ☐
- Moderato ☐
- Grave ☐
- Grado 4 ☐

C13.

Hai avuto astenia?

Giorno 6

Lieve: non interferisce con l'attività; moderato: una limitata interferenza con l'attività; grave: impedisce l'attività quotidiana; grado 4: visita al pronto soccorso o ricovero in ospedale per grave affaticamento

- Nessuno ☐
- Lieve ☐
- Moderato ☐
- Grave ☐
- Grado 4 ☐

C14.

Hai avuto astenia?

Giorno 7

Lieve: non interferisce con l'attività; moderato: una limitata interferenza con l'attività; grave: impedisce l'attività quotidiana; grado 4: visita al pronto soccorso o ricovero in ospedale per grave affaticamento

Nessuno ☐

Lieve ☐

Moderato ☐

Grave ☐

Grado 4 ☐

C15.

Hai avuto brividi?

Giorno 1

No ☐

Sì ☐

C16.

Hai avuto brividi?

Giorno 2

No ☐

Sì ☐

C17.

Hai avuto brividi?

Giorno 3

No ☐

Sì ☐

C18.

Hai avuto brividi?

Giorno 4

No ☐

Sì ☐

LimeSurvey

C19.

Hai avuto brividi?

Giorno 5

No ☐

Sì ☐

C20.

Hai avuto brividi?

Giorno 6

No ☐

Sì ☐

C21.

Hai avuto brividi?

Giorno 7

No ☐

Sì ☐

C22.

Hai avuto cefalea?

Giorno 1

Lieve: non interferisce con l'attività; moderato: una limitata interferenza con l'attività; grave: impedisce l'attività quotidiana; grado 4: visita al pronto soccorso o ricovero in ospedale per forte mal di testa

Grado 4 ☐

Grave ☐

Moderato ☐

Lieve ☐

Nessuno ☐

C23.

Hai avuto cefalea?

Giorno 2

Lieve: non interferisce con l'attività; moderato: una limitata interferenza con l'attività; grave: impedisce l'attività quotidiana; grado 4: visita al pronto soccorso o ricovero in ospedale per forte mal di testa

Grado 4 ☐

Grave ☐

Moderato ☐

Lieve ☐

Nessuno ☐

C24.

Hai avuto cefalea?

Giorno 3

Lieve: non interferisce con l'attività; moderato: una limitata interferenza con l'attività; grave: impedisce l'attività quotidiana; grado 4: visita al pronto soccorso o ricovero in ospedale per forte mal di testa

Grado 4 ☐

Grave ☐

Moderato ☐

Lieve ☐

Nessuno ☐

C25.

Hai avuto cefalea?

Giorno 4

Lieve: non interferisce con l'attività; moderato: una limitata interferenza con l'attività; grave: impedisce l'attività quotidiana; grado 4: visita al pronto soccorso o ricovero in ospedale per forte mal di testa

Grado 4 ☐

Grave ☐

Moderato ☐

Lieve ☐

Nessuno ☐

C26.

Hai avuto cefalea?

Giorno 5

Lieve: non interferisce con l'attività; moderato: una limitata interferenza con l'attività; grave: impedisce l'attività quotidiana; grado 4: visita al pronto soccorso o ricovero in ospedale per forte mal di testa

Grado 4 ☐

Grave ☐

Moderato ☐

Lieve ☐

Nessuno ☐

LimeSurvey

C27.

Hai avuto cefalea?

Giorno 6

Lieve: non interferisce con l'attività; moderato: una limitata interferenza con l'attività; grave: impedisce l'attività quotidiana; grado 4: visita al pronto soccorso o ricovero in ospedale per forte mal di testa

- Grado 4 ☐
- Grave ☐
- Moderato ☐
- Lieve ☐
- Nessuno ☐

C28.

Hai avuto cefalea?

Giorno 7

Lieve: non interferisce con l'attività; moderato: una limitata interferenza con l'attività; grave: impedisce l'attività quotidiana; grado 4: visita al pronto soccorso o ricovero in ospedale per forte mal di testa

- Grado 4 ☐
- Grave ☐
- Moderato ☐
- Lieve ☐
- Nessuno ☐

C29.

Hai avuto mialgie/artralgie?

Giorno 1

Lieve: non interferisce con l'attività; moderato: una limitata interferenza con l'attività; grave: impedisce l'attività quotidiana; grado 4: visita al pronto soccorso o ricovero in ospedale per forte dolore muscolare o forte dolore alle articolazioni

- Lieve ☐
- Nessuno ☐
- Grado 4 ☐
- Grave ☐
- Moderato ☐

C30.

Hai avuto mialgie/artralgie?

Giorno 2

Lieve: non interferisce con l'attività; moderato: una limitata interferenza con l'attività; grave: impedisce l'attività quotidiana; grado 4: visita al pronto soccorso o ricovero in ospedale per forte dolore muscolare o forte dolore alle articolazioni

- Lieve ☐
- Nessuno ☐
- Grado 4 ☐
- Grave ☐
- Moderato ☐

C31.

Hai avuto mialgie/artralgie?

Giorno 3

Lieve: non interferisce con l'attività; moderato: una limitata interferenza con l'attività; grave: impedisce l'attività quotidiana; grado 4: visita al pronto soccorso o ricovero in ospedale per forte dolore muscolare o forte dolore alle articolazioni

- Lieve ☐
- Nessuno ☐
- Grado 4 ☐
- Grave ☐
- Moderato ☐

C32.

Hai avuto mialgie/artralgie?

Giorno 4

Lieve: non interferisce con l'attività; moderato: una limitata interferenza con l'attività; grave: impedisce l'attività quotidiana; grado 4: visita al pronto soccorso o ricovero in ospedale per forte dolore muscolare o forte dolore alle articolazioni

- Lieve ☐
- Nessuno ☐
- Grado 4 ☐
- Grave ☐
- Moderato ☐

LimeSurvey

C33.

Hai avuto mialgie/artralgie?

Giorno 5

Lieve: non interferisce con l'attività; moderato: una limitata interferenza con l'attività; grave: impedisce l'attività quotidiana; grado 4: visita al pronto soccorso o ricovero in ospedale per forte dolore muscolare o forte dolore alle articolazioni

- Lieve ☐
- Nessuno ☐
- Grado 4 ☐
- Grave ☐
- Moderato ☐

C34.

Hai avuto mialgie/artralgie?

Giorno 6

Lieve: non interferisce con l'attività; moderato: una limitata interferenza con l'attività; grave: impedisce l'attività quotidiana; grado 4: visita al pronto soccorso o ricovero in ospedale per forte dolore muscolare o forte dolore alle articolazioni

- Lieve ☐
- Nessuno ☐
- Grado 4 ☐
- Grave ☐
- Moderato ☐

C35.

Hai avuto mialgie/artralgie?

Giorno 7

Lieve: non interferisce con l'attività; moderato: una limitata interferenza con l'attività; grave: impedisce l'attività quotidiana; grado 4: visita al pronto soccorso o ricovero in ospedale per forte dolore muscolare o forte dolore alle articolazioni

- Lieve ☐
- Nessuno ☐
- Grado 4 ☐
- Grave ☐
- Moderato ☐

LimeSurvey

C36.

Hai avuto sintomi gastrointestinali?

Giorno 1

Lieve: vomitare 1 o 2 volte in 24 ore e/o 2 o 3 feci molle in 24 ore; moderato: vomitare >2 volte in 24 ore e/o 4 o 5 feci molle in 24 ore; grave: richiede idratazione endovenosa e/o 6 o più feci molle in 24 ore; grado 4: visita al pronto soccorso o ricovero ospedaliero

- Grado 4 ☐
- Grave ☐
- Moderato ☐
- Lieve ☐
- Nessuno ☐

C37.

Hai avuto sintomi gastrointestinali?

Giorno 2

Lieve: vomitare 1 o 2 volte in 24 ore e/o 2 o 3 feci molle in 24 ore; moderato: vomitare >2 volte in 24 ore e/o 4 o 5 feci molle in 24 ore; grave: richiede idratazione endovenosa e/o 6 o più feci molle in 24 ore; grado 4: visita al pronto soccorso o ricovero ospedaliero

- Grado 4 ☐
- Grave ☐
- Moderato ☐
- Lieve ☐
- Nessuno ☐

C38.

Hai avuto sintomi gastrointestinali?

Giorno 3

Lieve: vomitare 1 o 2 volte in 24 ore e/o 2 o 3 feci molle in 24 ore; moderato: vomitare >2 volte in 24 ore e/o 4 o 5 feci molle in 24 ore; grave: richiede idratazione endovenosa e/o 6 o più feci molle in 24 ore; grado 4: visita al pronto soccorso o ricovero ospedaliero

- Grado 4 ☐
- Grave ☐
- Moderato ☐
- Lieve ☐
- Nessuno ☐

LimeSurvey

C39.

Hai avuto sintomi gastrointestinali?

Giorno 4

Lieve: vomitare 1 o 2 volte in 24 ore e/o 2 o 3 feci molle in 24 ore; moderato: vomitare >2 volte in 24 ore e/o 4 o 5 feci molle in 24 ore; grave: richiede idratazione endovenosa e/o 6 o più feci molle in 24 ore; grado 4: visita al pronto soccorso o ricovero ospedaliero

- Grado 4 ☐
- Grave ☐
- Moderato ☐
- Lieve ☐
- Nessuno ☐

C40.

Hai avuto sintomi gastrointestinali?

Giorno 5

Lieve: vomitare 1 o 2 volte in 24 ore e/o 2 o 3 feci molle in 24 ore; moderato: vomitare >2 volte in 24 ore e/o 4 o 5 feci molle in 24 ore; grave: richiede idratazione endovenosa e/o 6 o più feci molle in 24 ore; grado 4: visita al pronto soccorso o ricovero ospedaliero

- Grado 4 ☐
- Grave ☐
- Moderato ☐
- Lieve ☐
- Nessuno ☐

C41.

Hai avuto sintomi gastrointestinali?

Giorno 6

Lieve: vomitare 1 o 2 volte in 24 ore e/o 2 o 3 feci molle in 24 ore; moderato: vomitare >2 volte in 24 ore e/o 4 o 5 feci molle in 24 ore; grave: richiede idratazione endovenosa e/o 6 o più feci molle in 24 ore; grado 4: visita al pronto soccorso o ricovero ospedaliero

- Grado 4 ☐
- Grave ☐
- Moderato ☐
- Lieve ☐
- Nessuno ☐

LimeSurvey

C42.

Hai avuto sintomi gastrointestinali?

Giorno 7

Lieve: vomitare 1 o 2 volte in 24 ore e/o 2 o 3 feci molle in 24 ore; moderato: vomitare >2 volte in 24 ore e/o 4 o 5 feci molle in 24 ore; grave: richiede idratazione endovenosa e/o 6 o più feci molle in 24 ore; grado 4: visita al pronto soccorso o ricovero ospedaliero

Grado 4 ☐

Grave ☐

Moderato ☐

Lieve ☐

Nessuno ☐

C43.

Hai utilizzato farmaci antipiretici o antidolorifici?

Giorno 1

No ☐

Sì ☐

C44.

Hai utilizzato farmaci antipiretici o antidolorifici?

Giorno 2

No ☐

Sì ☐

C45.

Hai utilizzato farmaci antipiretici o antidolorifici?

Giorno 3

No ☐

Sì ☐

C46.

Hai utilizzato farmaci antipiretici o antidolorifici?

Giorno 4

No ☐

Sì ☐

C47.

Hai utilizzato farmaci antipiretici o antidolorifici?

Giorno 5

No ☐

Sì ☐

C48.

Hai utilizzato farmaci antipiretici o antidolorifici?

Giorno 6

No ☐

Sì ☐

C49.

Hai utilizzato farmaci antipiretici o antidolorifici?

Giorno 7

No ☐

Sì ☐

Sezione D: Sezione 2 - Vaccino COVID-19 mRNA: EVENTI AVVERSI RARI

D1.

Hai avuto linfoadenomegalia?

Giorno 1

No ☐

Sì ☐

D2.

Hai avuto linfoadenomegalia?

Giorno 2

No ☐

Sì ☐

D3.

Hai avuto linfoadenomegalia?

Giorno 3

No ☐

Sì ☐

D4.

Hai avuto linfoadenomegalia?

Giorno 4

No ☐

Sì ☐

D5.

Hai avuto linfoadenomegalia?

Giorno 5

No ☐

Sì ☐

D6.

Hai avuto linfoadenomegalia?

Giorno 6

No ☐

Sì ☐

D7.

Hai avuto linfoadenomegalia?

Giorno 7

No ☐

Sì ☐

D8.

Hai avuto sintomi a carico del sistema nervoso?

Giorno 1

Sì ☐

No ☐

D9.

Hai avuto sintomi a carico del sistema nervoso?

Giorno 2

Sì ☐

No ☐

D10.

Hai avuto sintomi a carico del sistema nervoso?

Giorno 3

Sì ☐

No ☐

D11.

Hai avuto sintomi a carico del sistema nervoso?

Giorno 4

Sì ☐

No ☐

D12.

Hai avuto sintomi a carico del sistema nervoso?

Giorno 5

Sì ☐

No ☐

D13.

Hai avuto sintomi a carico del sistema nervoso?

Giorno 6

Sì ☐

No ☐

LimeSurvey

D14.

Hai avuto sintomi a carico del sistema nervoso?

Giorno 7

Sì ☐

No ☐

LimeSurvey

IMPORTANTE: In caso di comparsa di reazioni insorte in periodo successivo ai 7 giorni dalla vaccinazione e/o di altre manifestazioni cliniche non ricomprese tra quelle precedentemente riportate a partire dal giorno dell'immunizzazione, si prega di contattare il Servizio di Sorveglianza Sanitaria del personale universitario.

I Servizi di Sorveglianza Sanitaria-Medicina del Lavoro e di Igiene di UniGe restano a completa disposizione, per quanto di competenza, per continuare a garantire l'attività d'informazione e consulenza.

Contatti

Servizio di Sorveglianza Sanitaria di Ateneo-Medicina del Lavoro Largo Rosanna Benzi 10, 16132 Genova Padiglione 3 Email: uos.sorveglianzasanitaria2@hsanmartino.it - sorvsanitaria@unige.it

Medici Specialisti e Medici Competenti: Prof. Paolo Durando - durando@unige.it; Dott.ssa Nicoletta Debarbieri - nicoletta.debarbieri@hsanmartino.it; Prof. Guglielmo Dini - guglielmo.dini@unige.it

Servizio di Igiene Largo Rosanna Benzi 10, 16132 Genova Padiglione 3 Email: igiene.segreteria@hsanmartino.it - ambulatorio.vaccinazioni@hsanmartino.it

Medici Specialisti: Prof. Giancarlo Icardi - icardi@unige.it; Dott.ssa Bianca Bruzzone - bianca.bruzzone@hsanmartino.it; Prof. Andrea Orsi - andrea.orsi@unige.it; Prof.ssa Laura Sticchi - laura.sticchi@unige.it

Grazie per aver partecipato all'indagine.
